# Optimal hash arrangement of tentacles in jellyfish

**DOI:** 10.1038/srep27347

**Published:** 2016-06-07

**Authors:** Takuya Okabe, Jin Yoshimura

**Affiliations:** 1 Graduate School of Integrated Science and Technology, Shizuoka University, Hamamatsu 432-8561, Japan; 2 Graduate School of Science and Technology, Shizuoka University, Hamamatsu 432-8561, Japan; 3 Marine Biosystems Research Center, Chiba University, Uchiura, Kamogawa, Chiba 299-5502, Japan; 4 Department of Environmental and Forest Biology, State University of New York College of Environmental Science and Forestry, Syracuse, NY13210, USA

## Abstract

At first glance, the trailing tentacles of a jellyfish appear to be randomly arranged. However, close examination of medusae has revealed that the arrangement and developmental order of the tentacles obey a mathematical rule. Here, we show that medusa jellyfish adopt the best strategy to achieve the most uniform distribution of a variable number of tentacles. The observed order of tentacles is a real-world example of an optimal hashing algorithm known as Fibonacci hashing in computer science.

A century ago, after investigating several hundred specimens of 0.6–30 mm in diameter, Kinoshita remarked that the tentacles and other organs of the jellyfish *Gonionemus vertens* are arranged in a regular order^1^,^2^. Without exception, these organs in each quadrant are arranged clockwise in the order 1, 6, 3, 8, 5, 2, 7, 4, 9, 1, as numbered in the sequence of development and viewed from below the umbrella. Komai and Yamazi have confirmed Kinoshita’s observation for approximately 60 specimens of *G. vertens* of 5–21 mm in diameter and with no more than 20 tentacles in each quadrant^3^. The authors have found that the size of these tentacles is nearly always in the order 1, 14, 6, 19, 11, 3, 16, 8, 13, 5, 18, 10, 2, 15, 7, 20, 12, 4, 17, 9, 1 and that exceptions to this pattern, if any, are found only among 11 to 20. This tendency has also been found in other hydrozoan jellyfishes including *Olindias formosus* (Fig. 1a), in which not only the number of the organs but also the number of radial canals are highly variable (Fig. 1b,c)^1^,^2^,^3^,^4^. Most importantly, the order conforms exactly to the arrangement commonly found in phyllotaxis^5^, i.e., the arrangement in which the organs are placed with constant intervals of angle equal to 137.5° (Fig. 1d)^3^,^4^.

Jellyfish have *N*-fold rotational symmetry^1^, i.e., a specimen looks the same after rotation by an angle of 360°/*N*. The following results do not depend on *N*, and we use a tetramerous sample (*N* = 4) for purposes of illustration. The number of organs in each part of *N* units is denoted as *T*. We make the biological assumption that the jellyfish is genetically programmed to generate organs successively at constant intervals of angle. In each unit, this angle is denoted as *α*/*N* (Fig. 1d). Thus, the angle 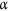 is defined as the angle between successive organs (*α*/*N*) multiplied by the number (*N*) of equivalent units. We assume that this angle (*α*) has been optimised during the course of evolution. The use of *α* in place of the actual angle *α*/*N* is only for the sake of presentation.

Each unit subtends an angle of 360°/*N*, which is divided into *T* intervals by *T* organs. As indicated in the bottom of Fig. 1d, in an ordinal sequence, the angle of the *i*-th interval is denoted as *θ*_*i*_ (*i* = 1, 2, …, *T*). The mean and variance of *θ*_*i*_ are given by 360°/*N*/*T* and


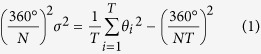


In this equation, the variance *σ*^2^ of the interval angles is defined such that it is independent of *N*. To signify that *σ*^2^ is determined by the angle *α* and *T*, it is denoted as *σ*^2^(*α*, *T*). The smaller the value of *σ*^2^(*α*, *T*), the more evenly the distribution of organs. As the number of organs (*T*) is not constant but increases as the medusa grows, we regard an average over *T* of *σ*^2^(*α*, *T*) as the inverse of a fitness function,


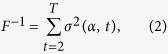


or regard *F*^−1^ as unfitness to be minimised. Weighted summation can be used without affecting the main point (Supplementary Figs S1 and S2).

Figure 2a shows the fitness function *F*(*α*) for *T* = 5, 10 and 15. The observed angle of *α* = 137.5° lies close to the peak of the fitness landscape. The averaging operation in equation (2) plays a role in setting the peak at *α* = 137.5°. If the number of organs (*T*) is not variable, the variance *σ*^2^ has the minimum value zero when *α* is a multiple of 360°/*T*. Indeed, Fig. 2b shows that *σ*^2^ for *T* = 5 vanishes at *α* = 360° × 1/5 = 72° and 360° × 2/5 = 144°. Remarkably enough, the observed order (1, 14, 6, 19, etc.) and the reported irregularity indicate that *α* is adjusted as accurately as 137.5 ± 2° (Table 1 and Supplementary Fig. S3). This fine-tuning is consistent with the enlarged plot for *T* = 20 in Fig. 2c. Thus, medusa jellyfish are very finely adapted to make use of the optimal arrangement of organs.

Interestingly, the above result corresponds with a mathematical theorem used for a class of search methods commonly known as hashing techniques in computer programming. In terms of our language, the theorem reads as follows (p. 518 of ref. 6):

## Theorem S.

*When the organs are placed at* 0, 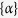, 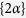, 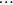, 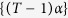 *in the angle range of* [*0, 360°*], *the T gaps formed by the T organs have at most three different sizes, where* 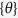 *denotes the remainder after the division of θ by 360°* (*e.g.*, 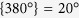)*. Moreover, the next organ* 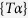 *will arise in one of the largest gaps*.

Figure 3 illustrates this theorem for *α*/*N* = 137.5°/4 (Fig. 3a) and *α*/*N* = 160°/4 (Fig. 3b). The gaps of a given angle are created and destroyed in a first-in, first-out manner, i.e., a new organ arises in the oldest of the largest gaps. This rule holds true for any value of *α*. The uniqueness of 137.5° (and its mirror-image equivalent 222.5°) is in that this angle leads to the “most uniformly distributed” gaps^6^ in the sense that this ideal angle divides every gap in the ratio of the golden mean 1:1.618. All the other angles will cause a *bad break*^6^ with a division ratio greater than 1:2. In fact, the observed order of organs is simply a real-world example of a hashing algorithm known as *Fibonacci hashing*, in which collisions of organs are avoided most effectively (Fig. 37 of ref. 6).

**Figure 2:**
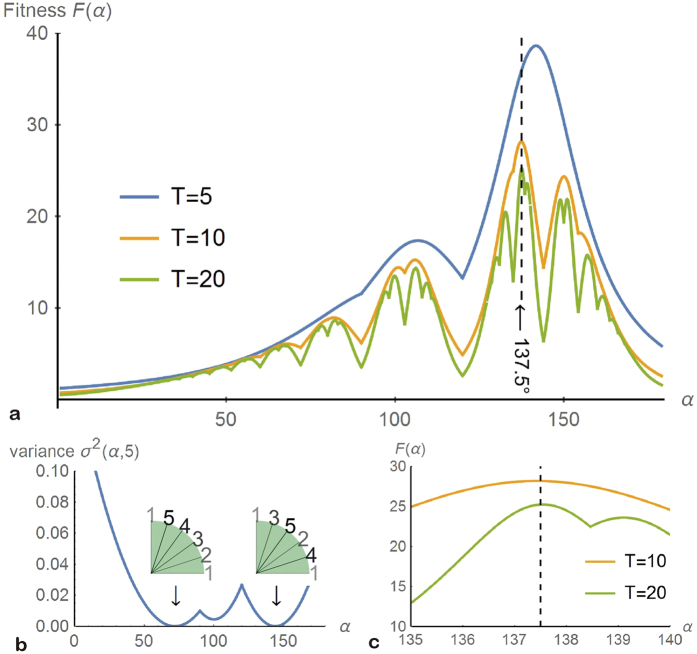
The 137.5*°* angle is the fittest angle. **(a)** *F* (*α*) for *T* = 5, 10, and 20. **(b)** *σ*^2^(*α*) for *T* = 5. **(c)** Enlargement of (**a**).

**Figure 3:**
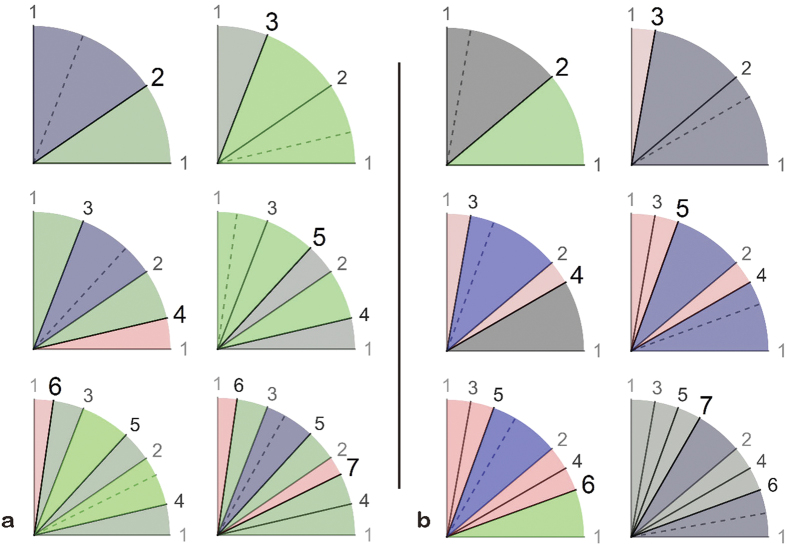
Every new organ occurs in a largest gap. In each panel, the number in bold is *T* (=2, 3, 4, 5, 6, 7), a dashed line denotes the next organ (*T* + 1), and gaps of different sizes are shown in different colours. **(a**) The optimal case of *α*/*N* = 137.5°/4. **(b)** A general case of *α*/*N* = 160°/4, in which organs are ordered as shown in the last row of Table 1. Bad breaks (with division ratios > 2) will occur unless *α* = 137.5° (ref. 6).

Quotation marks are used for the “most uniformly distributed” gaps to allow for an important, exceptional case in which *α* happens to be a rational number, i.e., a ratio of integers. In this case, the smallest gaps can be zero angles because organs overlap (or collide) when the tentacle number 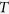
is larger than the denominator of the rational number *α* (collision occurs if *T* > 5 in the case of *α* = 360° × 2/5 = 144°). For a fixed number of organs, even distribution with a rational number of *α* is trivially the most uniform distribution (Fig. 2b). Accordingly, the most uniform distribution does not necessarily indicate a 137.5° angle. A variable number of organs is essential for explaining the prevalence of 137.5°. Medusa jellyfish adopt the best strategy to always achieve the most uniform distribution of a variable number of organs that arise one after another.

The purpose of the present article was to provide an evolution-theoretical explanation for the little-known findings of the presence of 137.5° in the order of jellyfish tentacles. The explanation is based on the following plausible assumptions at different biological levels: (i) at the level of organogenesis, an indeterminate number of organs successively arise at constant intervals of angle, and (ii) at the level of organisms, natural selection favours a well-balanced arrangement. As in the discipline of theoretical morphology^7^, the present approach is theoretical in that, to find an optimal arrangement, theoretical possibilities are hypothetically compared with each other. The generality of this approach is both its strength and its weakness. Its weakness is that optimality is not empirically testable without preparing a control group with non-optimal arrangement. Its strength is that it is independent of practical details. The first point (i) is a developmental constraint, whereas there are proximate and ultimate factors for (ii). As an ultimate factor, a uniform distribution is most likely to confer a survival advantage for foraging and locomotion of jellyfish for the very reasons directly related to the functional significance of dispersed appendages. From the viewpoint of fluid mechanics, uniform arrangement of tentacles should contribute to the planktonic mode of life of jellyfish as it enhances viscous forces experienced by the body. Thus, to the extent that a plural number of tentacles are functional to the animal, they are utilized most effectively when they are dispersed away from each other. The selective pressure due to this collective factor is operative insofar as their total effect is more than a simple sum of parts, or to the extent that the arrangement of organs matters. Concerning (i) and a proximate factor for (ii), it is instructive to compare with leaf arrangement in plant^5^. The overall processes of growth and formation of lateral organs in a hydra and a plant are strictly comparable^8^. In plant phyllotaxis, the empirical rule that new leaves always arise in the largest gap between those already present is known as Hofmeister’s rule^9^. This rule is interpreted as a result of a physical or chemical influence of neighbouring organs^5^,^8^,^10^,^11^. The mutual influence to avoid collision of organs serves as a proximate factor for (ii). In plants, the ultimate cause of collision avoidance should be sought in leaf exposure to light^12^ or in the vascular structure of leaves^13^. Optimisation in terms of the fitness in equation (2) has much in common with the concept of geometric mean fitness, which is used as a measure of a long-term survival probability under stochastic environments^14^. It is important that either one of the constraints (i) and (ii), if confirmed, does not lead to 137.5°. The peculiarity of this trait is that it is not optimal for a particular individual at a particular time. The 137.5° angle is the optimal compromise, or “the golden mean”, between the conflicting constraints^13^. In this general sense, the evolution of the 137.5° angle in plants and jellyfish is a hitherto unnoticed example of parallel evolution, although the habitat environment and the constraint factors are specifically different in the taxa of different lineages.

## Additional Information

**How to cite this article**: Okabe, T. and Yoshimura, J. Optimal hash arrangement of tentacles in jellyfish. *Sci. Rep*. **6**, 27347; doi: 10.1038/srep27347 (2016).

## Supplementary Material

Supplementary Information

## Figures and Tables

**Figure 1:**
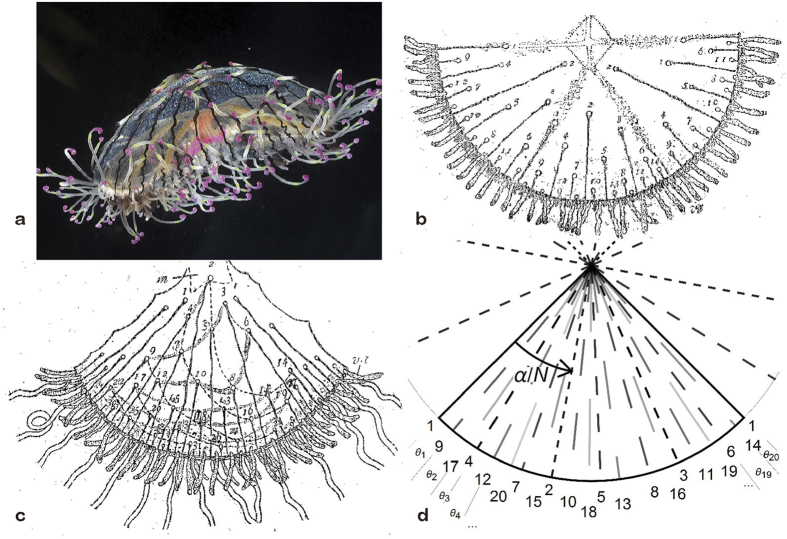
Tentacles of jellyfish are arranged in accordance with a mathematical rule. **(a)** Flower hat jelly (*Olindias formosus*). **(b)** Arrangement of exumbrellar tentacles in three sextants of a hexamerous (*N* = 6) specimen of *O. formosus*. The tentacles are numbered up to *T* = 14 in the order of development. **(c)** Arrangement of exumbrellar tentacles in a quadrant of a tetramerous (*N* = 4) specimen of *O. formosus* (numbered up to *T* = 31). **(d)** The theoretical arrangement in which consecutive organs are placed at constant intervals of *α/N* = 137.5*°/*4 conforms to the observed order 1, 14, 6, 19, 11, etc. (*T* = 20, *N* = 4). Photo by Port of Nagoya public aquarium. Drawings reproduced from Komai and Yamazi^3^ (not copyrighted).

**Table 1:** The order of organs theoretically expected for a given range of values of *α*.

*α*/360°(*α*)	Order
0–1/8	1, 9, 8, 7, 6, 5, 4, 3, 2, 1
1/8–1/7	1, 8, 7, 6, 5, 4, 3, 2, 9, 1
1/7–1/6	1, 7, 6, 5, 4, 3, 9, 2, 8, 1
1/6–1/5	1, 6, 5, 4, 9, 3, 8, 2, 7, 1
1/5–1/4	1, 5, 9, 4, 8, 3, 7, 2, 6, 1
1/4–2/7	1, 8, 4, 7, 3, 6, 2, 9, 5, 1
2/7–1/3	1, 4, 7, 3, 6, 9, 2, 5, 8, 1
1/3–3/8	1, 9, 6, 3, 8, 5, 2, 7, 4, 1
**3/8–8/21 (135–137.1°)**	**1, 22, 14, 6, 19, 11, 3, 16, 8, 21, 13, 5, 18, 10, 2, 15, 7, 20, 12, 4, 17, 9, 1**
**8/21–5/13 (137.1–138.5°)**	**1, 14, 6, 19, 11, 3, 16, 8, 21, 13, 5, 18, 10, 2, 15, 7, 20, 12, 4, 17, 9, 22, 1**
5/13–2/5	1, 6, 11, 3, 8, 13, 5, 10, 2, 7, 12, 4, 9, 14, 1
2/5–3/7	1, 8, 3, 5, 7, 2, 9, 4, 6, 1
3/7–1/2	1, 3, 5, 7, 9, 2, 4, 6, 8, 1

As shown in bold rows, the order 1, 14, 6, 19, etc. is obtained only if *α* is larger than 135° and smaller than 138.5°.
