# Supplementary material for: Optimal hash arrangement of tentacles in jellyfish
Source: Sci Rep. 2016 Jun 7;6:27347. doi: 10.1038/srep27347 (PMC4895151; doi:10.1038/srep27347)
Supplement: Supplementary Information [file srep27347-s1.pdf]

Supplementary Information for

Optimal hash arrangement of tentacles in jellyfish by

Takuya Okabe and Jin Yoshimura

Supplementary Note

Supplementary Figures S1-3

Supplementary Note

To see the robustness and reproducibility of the main results, Fig. S1 plots F for T from 2 to 9. This figure and Fig. 2a indicate that the main peak at $\alpha = 137.5°$ do not depend on the manner in which the terms in Eq. (2) are averaged. To see the effect of the first term $\sigma^2(\alpha,2)$, Eq. (2) is written as

$$F^{-1} = c\sigma^2(\alpha,2) + \sum_{t=3}^{T} \sigma^2(\alpha,t).$$

The first term is separated and multiplied with a factor c. For T=10, F for $c = 1$, 0.2 and 0.02 are plotted in Fig. S2. The curve for c=1 is the same as the curve for T=10 in Fig.2. Figures S1 and S2 indicate that the use of weighted average in place of Eq. (2) may affect the relative height of suboptimal peaks. The most prominent peak at 137.5° is overtaken by a shoulder peak at 99.5° if the term $\sigma^2(\alpha,2)$ is suppressed by a factor as small as c=0.02. The absence of the term $\sigma^2(\alpha,2)$ means that the number of tentacles in each unit begins with three at a time. In plant phyllotaxis, this secondary angle is rarely found in some species[5,13].

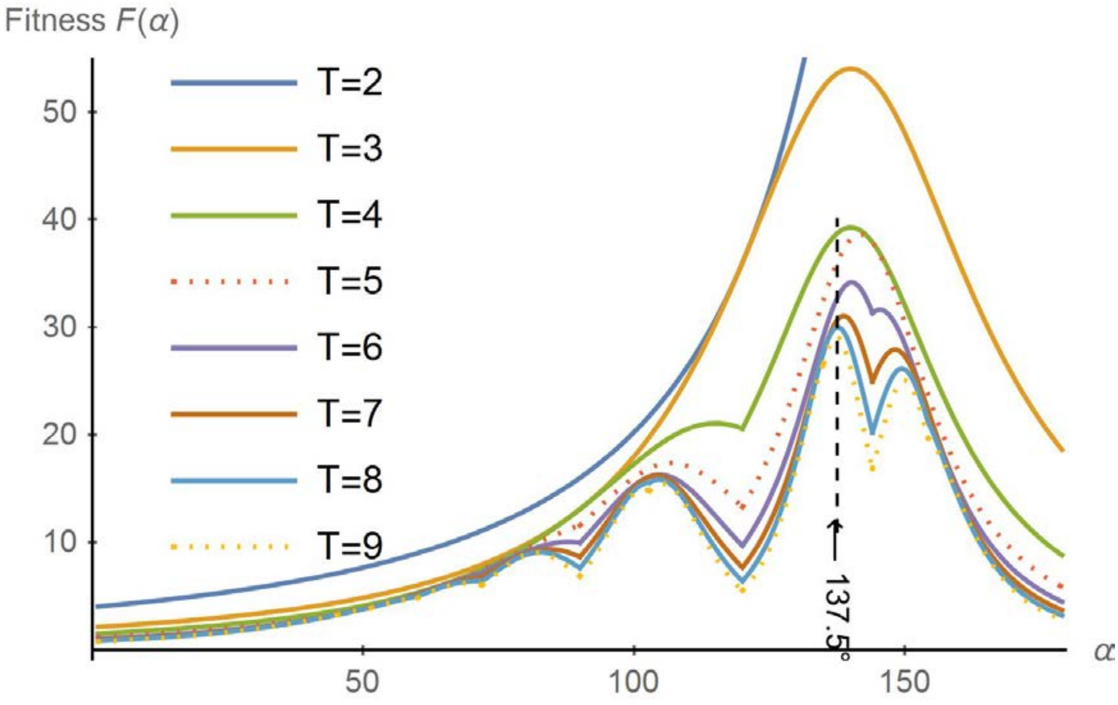

Supplementary Figure S1 | The peak at 137.5° is a robust result of averaging over the tentacle number *T*.

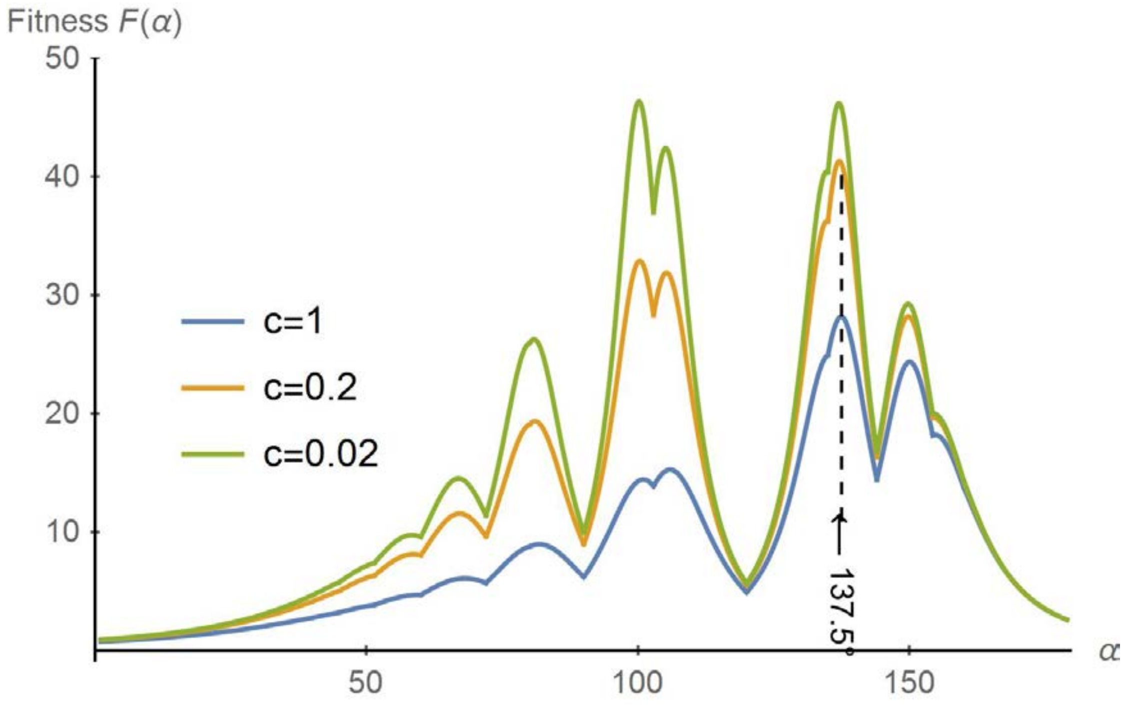

Supplementary Figure S2 | The peak at 137.5° gives way to a second peak at 99.5° if the contribution from the first term (*T*=2) is suppressed by a factor of *c*=0.02 (*T*=10).

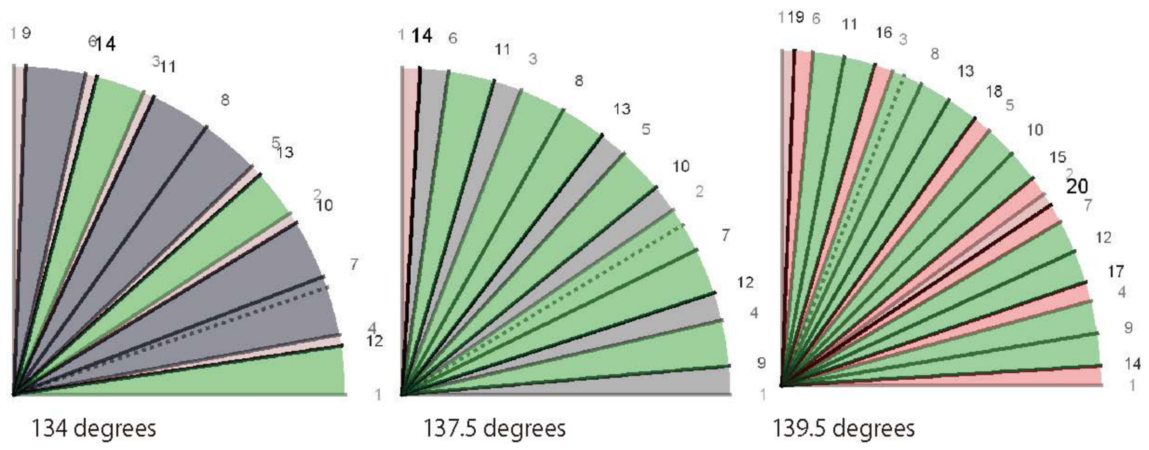

Supplementary Figure S3 | Different angles give different orders.
